# Establishment and Validation of a Ferroptosis-Related Gene Signature to Predict Overall Survival in Lung Adenocarcinoma

**DOI:** 10.3389/fgene.2021.793636

**Published:** 2022-01-14

**Authors:** Su Wang, Zhen Xie, Zenghong Wu

**Affiliations:** ^1^ Department of Emergency Medicine, Union Hospital, Tongji Medical College, Huazhong University of Science and Technology, Wuhan, China; ^2^ Department of Otorhinolaryngology, Union Hospital, Tongji Medical College, Huazhong University of Science and Technology, Wuhan, China; ^3^ Department of Infectious Diseases, Union Hospital, Tongji Medical College, Huazhong University of Science and Technology, Wuhan, China

**Keywords:** Lung Adenocarcinoma, ferroptosis, genes, immune infiltration, data mining

## Abstract

**Background:** Lung adenocarcinoma (LUAD) is the most common and lethal subtype of lung cancer. Ferroptosis, an iron-dependent form of regulated cell death, has emerged as a target in cancer therapy. However, the prognostic value of ferroptosis-related genes (FRGs)x in LUAD remains to be explored.

**Methods:** In this study, we used RNA sequencing data and relevant clinical data from The Cancer Genome Atlas (TCGA) dataset and Gene Expression Omnibus (GEO) dataset to construct and validate a prognostic FRG signature for overall survival (OS) in LUAD patients and defined potential biomarkers for ferroptosis-related tumor therapy.

**Results:** A total of 86 differentially expressed FRGs were identified from LUAD tumor tissues versus normal tissues, of which 15 FRGs were significantly associated with OS in the survival analysis. Through the LASSO Cox regression analysis, a prognostic signature including 11 FRGs was established to predict OS in the TCGA tumor cohort. Based on the median value of risk scores calculated according to the signature, patients were divided into high-risk and low-risk groups. Kaplan–Meier analysis indicated that the high-risk group had a poorer OS than the low-risk group. The area under the curve of this signature was 0.74 in the TCGA tumor set, showing good discrimination. In the GEO validation set, the prognostic signature also had good predictive performance. Functional enrichment analysis showed that some immune-associated gene sets were significantly differently enriched in two risk groups.

**Conclusion:** Our study unearthed a novel ferroptosis-related gene signature for predicting the prognosis of LUAD, and the signature may provide useful prognostic biomarkers and potential treatment targets.

## Introduction

Lung cancer is the leading cause of cancer-related lethality around the world, with almost 1.6 million deaths per year, and the 5-year survival rate still lags at below 20% (Uras et al., 2020). Lung adenocarcinoma (LUAD) represents the most common subtype of lung cancer, accounting for ∼50% of all cases (Chen et al., 2020). Some risk factors such as smoking, family history of lung cancer, aging, and virus infection have been implicated in the initiation and progression of LUAD (Nakhaie et al., 2020), and molecules and pathways mediating the occurrence and development of LUAD have been continuously investigated (Wang et al., 2020; Zhou et al., 2020). Unfortunately, more than 70% of cases are with advanced disease at diagnosis (Domagala-Kulawik and Trojnar, 2020). Moreover, despite neoplasm of the lowest stage, there is still a high risk of metastatic relapse after excision (Liljedahl et al., 2020). Over the last decades, the survival rate of patients with LUAD has been improved very little, although progress has been made in treatment (Ma et al., 2020). Therefore, it is vital to explore reliable and promising prognostic biomarkers for LUAD and identify valuable therapeutic targets. At present, several drugs have been reported to possess remarkable antitumor effects on LUAD *via* inducing autophagy and apoptosis (Liu et al., 2016; Cao et al., 2019). In addition, dynamic BH3 profiling is used to measure changes in chemotherapeutics-induced apoptotic signaling, and using BH3 mimetic drugs which increase mitochondrial apoptotic priming may enforce the apoptotic fate of LUAD cells (Montero et al., 2015; Sánchez-Rivera et al., 2021). However, the exploration of other forms of cell death to uncover new biomarkers and targets for LUAD is also urgently required.

Ferroptosis is a novel type of regulated cell death driven by the lethal levels of iron-dependent lipid hydroperoxide accumulation and has attracted much interest in recent years (Stockwell et al., 2017). Oxidative stress caused by excess iron is correlated with carcinogenesis which is considered a process of ferroptosis resistance as well as iron addiction, and both of them also occur in tumor cells (Toyokuni, 2016; Toyokuni et al., 2017). Due to the strong demand for iron to support rapid proliferation, tumor cells are vulnerable to the overload of iron and accumulation of reactive oxygen species (ROS), which in turn enables the ferroptosis‐mediated cancer therapy (Liang et al., 2019). Moreover, ferroptosis might enhance the antitumor effect of apoptosis inducer cisplatin, indicating that ferroptosis inducers may help overcome the resistance of cancer cells to traditional anticancer drugs (Roh et al., 2017; Wu et al., 2020). Increasing studies have explored the role of ferroptosis-related genes in lung cancer. Lai et al. found that overexpression of glutathione peroxidase 4 (*GPX4*) in lung cancer cells promoted proliferation but attenuated abnormalities specific to ferroptosis (Lai et al., 2019). The sensitivity of non-small cell lung cancer (NSCLC) cells to cysteine deprivation-induced ferroptosis could be regulated by nuclear factor-erythroid 2-like 2 (*NRF2*) *via* the FOCAD-FAK signaling pathway (Liu et al., 2020). Erastin-induced ROS promoted the upregulation and activation of *p53*, which contributed to the cytostatic and cytotoxic effects in lung cancer cells (Huang et al., 2018). Moreover, Ji et al. showed that highly expressed cystine-glutamate transporter (*SLC7A11*) mediated metabolic requirements during NSCLC progression and predicted a worse 5-year survival (Ji et al., 2018). A few previous studies have studied prognostic models related to ferroptosis (Jin et al., 2021; Wang et al., 2021). However, the role of a large number of ferroptosis-related genes in LUAD patients remains unclear and to systematically evaluate ferroptosis-related gene signature and its relationship with overall survival (OS) in LUAD is still needed. In this study, we used data extracted from The Cancer Genome Atlas (TCGA) database and Gene Expression Omnibus (GEO) database to construct and validate a prognostic signature of ferroptosis-related genes and assess their importance as biomarkers for ferroptosis‐mediated cancer therapy.

## Materials and Methods

### Data Collection

We obtained RNA-seq data of 54 normal samples and 497 LUAD samples, along with related clinical data, from the TCGA database (https://tcgadata.nci.nih.gov/tcga/, October 2020). The expression profiles and relevant clinical data of 462 tumor samples (GSE68465) were gained from the GEO portal (https://www.ncbi.nlm.nih.gov/geo/). A list of 259 (108 drivers; 69 suppressors; 111 markers) ferroptosis-related genes (FRGs) was gained from FerrDb (Zhou and Bao, 2020) and is shown in Supplementary Table S1.

### Identification of Prognosis-Related Differentially Expressed FRGs

The R package “limma” was used for screening out FRGs in the TCGA transcriptome data. To facilitate subsequent validation, we identified the shared FRGs between the TCGA and GEO datasets through intersecting the selected FRGs with gene expression profiles of GSE68465 *via* R package “limma” and “sva.” The shared FRGs and their expression with correction and standardization were extracted from the TCGA dataset and GEO dataset, respectively. In the TCGA cohort, among all shared FRGs, differentially expressed FRGs (DEFRGs) were identified between LUAD samples and normal samples, based on the cutoff threshold as |log2 fold change (FC)| >0.5 and adjusted *p*-value <0.05. A volcano map was conducted to visualize the DEFRGs. We explored the biological functions of the identified DEFRGs using Gene Ontology (GO) function and Kyoto Encyclopedia of Genes and Genomes (KEGG) analyses *via* the R language “ggplot2” package. Last, univariate Cox regression analysis was used to screen out the prognostic DEFRGs (PDEFRGs) that were significantly related to OS of patients in the TCGA LUAD dataset.

### Establishment and Validation of a Prognostic FRGs Signature

Based on the PDEFRG expression and survival data, a prognostic gene signature was established through the least absolute shrinkage and selection operator (LASSO) Cox regression analysis with R package called “glmnet.” FRGs with independent prognostic values were included in the signature, and the risk score of this signature was calculated as follows: score = sum (each gene’s expression × corresponding regression coefficient). According to the signature, the risk score for each LUAD patient was calculated. Based on the median value of all patients’ risk scores in the TCGA LUAD cohort, all patients were separated into a high-risk group and a low-risk group. The signature and identified median value were subsequently applied to the GEO cohort for validation, and all LUAD patients in the GEO dataset were also divided into two risk groups. A heatmap was used to visualize the distribution of clinicopathological features in the high-risk and low-risk groups. The correlation analysis with R package “ggpubr” and “limma” was used to explain the correlation between the risk scores and subgroups of clinicopathological characteristics. The survivals of the two groups of patients were analyzed through Kaplan–Meier (K–M) curve analysis with R packages “survival” and “survminer.” Univariate and multivariate Cox regression analysis were used to evaluate the association between risk score and prognosis. In addition, a nomogram was built to predict OS for clinical application, based on the results of the multivariate Cox analysis. The time-dependent receiver operating characteristic (ROC) curve was plotted to assess the predictive ability of the prognostic signature for 1-, 3-, and 5-year survival using R packages “timeROC” and “survival.”

### Enrichment analysis

Gene set enrichment analysis (GSEA) was performed according to the GSEA software (version 4.1.0) to explore the molecular mechanism and critical signaling pathways difference between the low-risk and high-risk groups. False discovery rate (FDR) < 0.25 and nominal *p* value < 0.05 were considered noteworthy. Moreover, we used the single-sample GSEA (ssGSEA) and R package “GSVA” to quantify activities of tumor-infiltrating immune cells between risk groups using 29 immune signatures and find different immune responses and functions.

### Online Database Analysis

Online databases were used to study different types of gene alterations in tumors and provide distinct prognostic values in LUAD patients. We utilized the cBioPortal (http://cbioportal.org) (Wu et al., 2019), an open-access site providing download, analysis, and visualization of large-scale cancer genomics datasets, to analyze the mutations of FRGs in the prognostic model. The kmplot (https://kmplot.com) online tool was performed to assess the impact of genes expression on survival of lung cancer.

### Statistical Analysis

R software (version 4.0.3) and SPSS (version 23.0) were used for all statistical analyses, and Strawberry Perl (version 5.32.0.1) was applied to data matrix and data processing. The unpaired Student’s t-test and the Wilcoxon test or Mann–Whitney *U*-test were performed to evaluate the normal distribution variables and the non-normal distribution variables, respectively. Categorical variables were tested with a chi-square or Fisher’s exact test. Univariate Cox, multivariate Cox, and LASSO Cox analyses were used to identify significant prognostic variables. The OS was analyzed by K–M analysis using a log-rank test. *p* value < 0.05 meant statistical significance.

## Results

### Identification of Prognosis-Related DEFRGs in LUAD Patients

**FIGURE 1:**
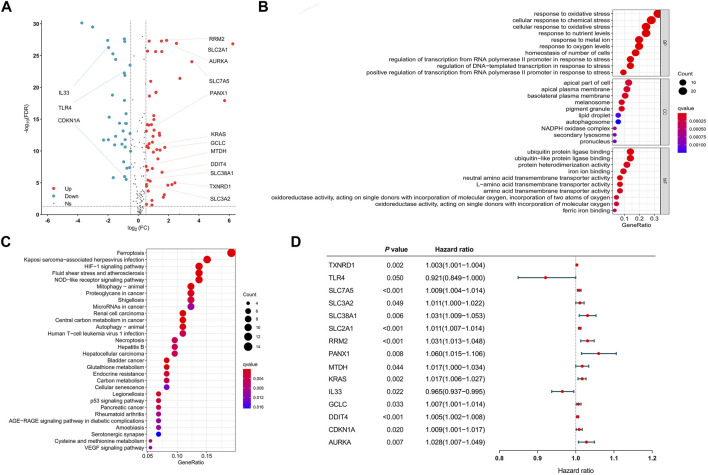
Visualization of differential ferroptosis-related genes. **(A)** Volcano map showing FRGs between normal and tumor tissues. **(B)** GO results of differentially expressed FRGs. **(C)** KEGG results of differentially expressed FRGs. **(D)** Forest plots showing the results of the univariate Cox regression analysis between DFRG expression and overall survival. GO: gene ontology. KEGG: Kyoto Encyclopedia of Genes and Genomes. FRGs: ferroptosis-related genes. DFRGs: differentially expressed ferroptosis-related genes.

After all genes were searched in sequence, 245 FRGs were identified to be expressed in the TCGA cohort. Next, we identified 210 FRGs shared between the TCGA samples and the GEO samples. Among these shared genes, 86 FRGs (52 upregulated genes and 34 downregulated genes) were differentially expressed between normal and tumor tissues and were used to model the prognostic signature for LUAD patients (Figure 1A and Supplementary Table S2). Biological processes (BP) of 86 DEFRGs were mainly enriched in response to oxidative stress and cellular response to chemical stress (Figure 1B). Cellular components (CC) were mainly enriched in the apical part of the cell and apical plasma membrane. Molecular functions (MF) were mainly enriched in iron ion binding and ubiquitin protein ligase binding. Ferroptosis, HIF-1 signaling pathway, and NOD-like receptor signaling pathway were significantly enriched in the KEGG pathway analysis (Figure 1C). Through the univariate Cox analysis, 15 DEFRGs (13 risk factors and two protective factors) were significantly associated with OS in the TCGA tumor cohort (Figure 1D).

### Development of the Prognostic FRG Signature

The 15 prognosis-related DEFRGs mentioned above were further analyzed by LASSO Cox regression analysis, and 11 FRGs were filtered to construct a prognostic model, including *CDKN1A*, *DDIT4*, *IL33*, *KRAS*, *MTDH*, *PANX1*, *RRM2*, *SLC2A1*, *SLC7A5*, *TLR4*, and *TXNRD1*. Finally, we established an 11-FRG signature to predict OS in the LUAD patients. The risk score formula was as follows: Risk score = (0.0049 × expression_*CDKN1A*_) + (0.0037 × expression_*DDIT4*_) - (0.0121 × expression_*IL33*_) + (0.0057 × expression_*KRAS*_) + (0.0089 × expression_*MTDH*_) + (0.0169 × expression_*PANX1*_) + (0.0060 × expression_*RRM2*_) + (0.0057 × expression_*SLC2A1*_) + (0.0041 × expression_*SLC7A5*_) - (0.0380 × expression_*TLR4*_) + (0.0003 × expression_*TXNRD1*_).

### Survival Results and Multivariate Examination

**FIGURE 2:**
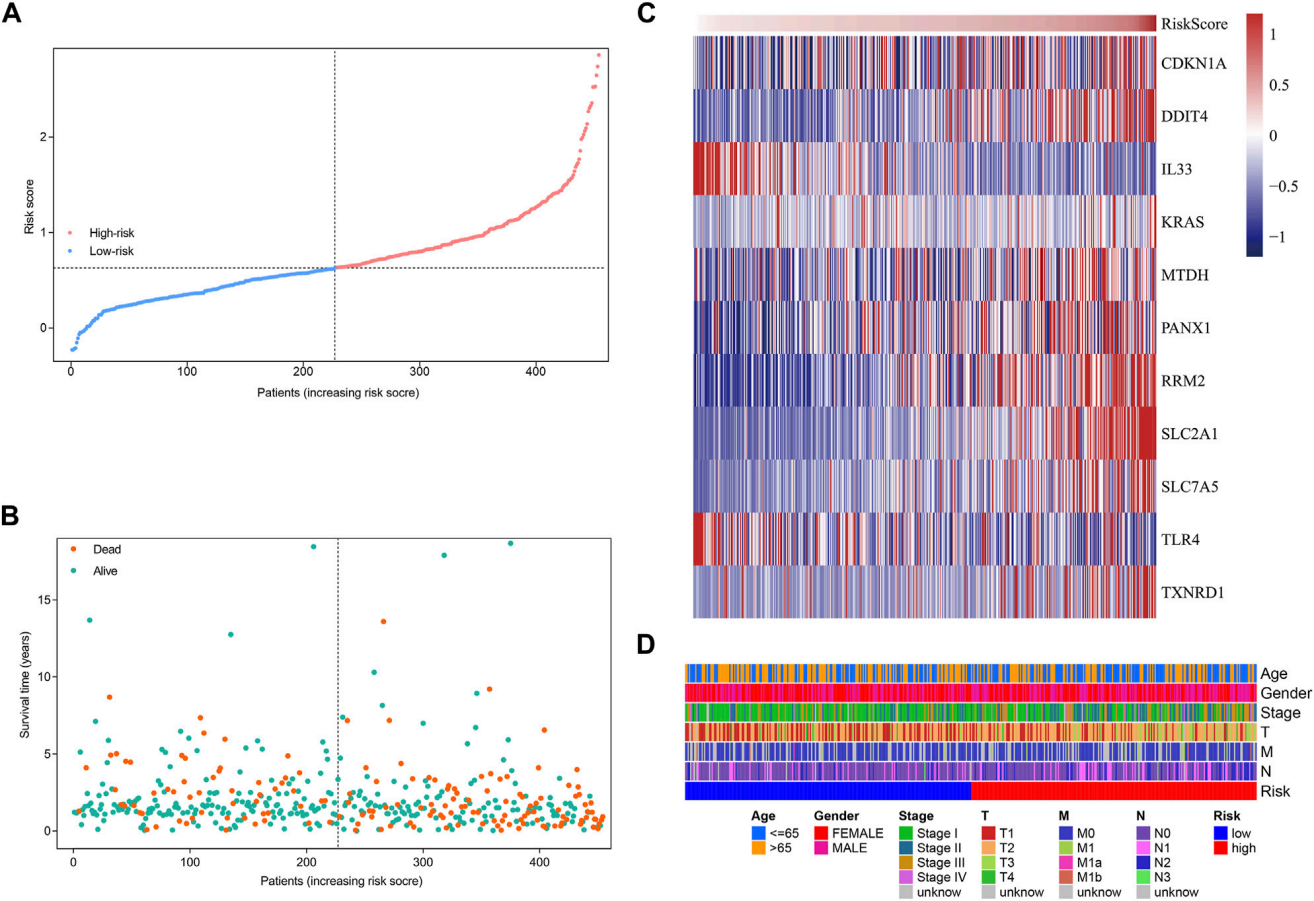
Characteristics of risk scores and ferroptosis-related gene signature in the TCGA tumor cohort. **(A)** The distribution and median value of the risk scores. **(B)** The distributions of survival status, survival time, and risk score. **(C)** Heatmap showing the expression of model genes in two risk groups. **(D)** Heatmap showing the distribution of clinicopathological features in two risk groups.

**FIGURE 3:**
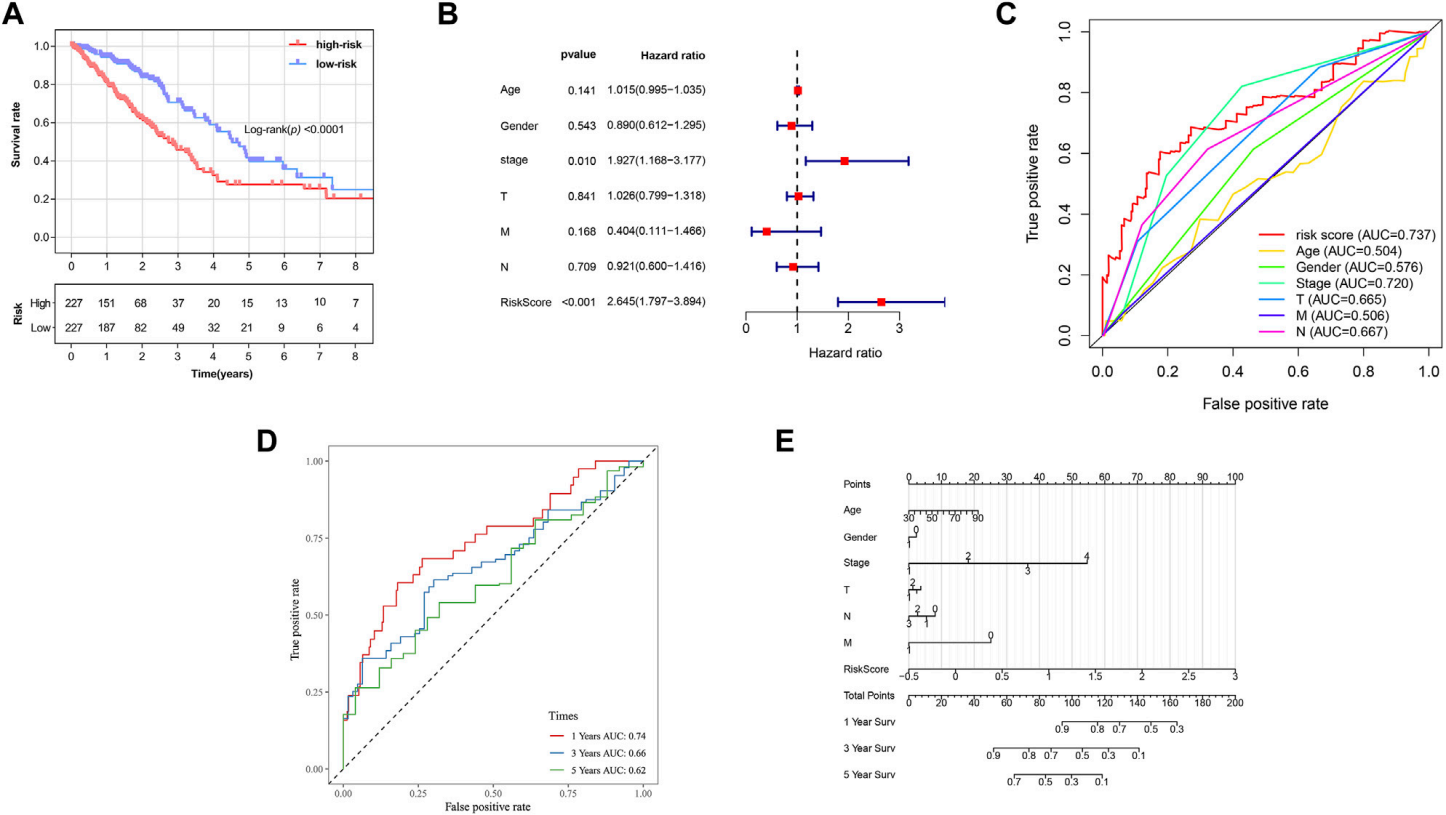
Prognostic analysis of ferroptosis-related gene signature in the TCGA tumor cohort. **(A)** Kaplan–Meier survival curve of the high- and low-risk groups. **(B)** Multivariate Cox regression analysis on overall survival. **(C)** ROC curves for predicting the overall survival of clinical factors and risk score. **(** **D)** ROC curves for predicting the 1-, 3-, and 5-year survival of risk score. **(** **E)** Nomogram for predicting overall survival. Gender: 1, male; 0, female. Surv: survival. ROC: receiver operating characteristic curve. AUC: area under the curve.

Based on the formula, we calculated the risk scores of LUAD patients in the TCGA dataset. According to the median risk score, the TCGA tumor cohort was divided into high-risk and low-risk groups, and each group was assigned 227 LUAD patients (Table 1). The impact of risk scores on risk level and survival, the expression of eleven prognostic-associated FRGs based on the risk scores, and the clinicopathological features in two risk groups are presented in Figures 2A–D. Comparisons of the risk scores among subgroups according to clinicopathological characteristics are shown in Supplementary Figures S1A–D, and there existed a significant correlation between risk scores and age, tumor stage, tumor size stage, and lymph node stage. To identify the prognostic difference between the risk groups, we performed a KM survival analysis and the results showed that the high-risk group had a significantly poorer outcome compared with the low-risk group (Figure 3A, *p* < 0.0001). The further performed multivariate Cox analysis demonstrated that the risk model was a significant prognostic predictor, independent of other clinical factors (Supplementary Table S3 and Figure 3B **,** *p* < 0.001). Moreover, the area under the ROC curve (AUC) of the risk score model was 0.737, which was higher than that of other clinical indices, showing a better prognostic prediction efficacy (Figure 3C). We also plotted the ROC curves to assess the efficiency of risk scores in predicting 1-, 3-, and 5-year survival, and the AUC was 0.74, 0.66, and 0.62, respectively (Figure 3D). In addition, the nomogram was constructed in combination of clinicopathological parameters and an 11-FRG signature (Figure 3E). Based on the score of each item in the nomogram, the total score could be calculated to predict the 1-, 3-, and 5-year survival rates of LUAD patients.

**TABLE 1:** Characteristics of two risk group patients in TCGA LUAD cohort.

Characteristics	TCGA-LUAD cohort		
	High risk	Low risk	*p*-value
	(*n* = 227)	(*n* = 227)	
Age, years	63.7 (38-88)	66.1 (33-87)	0.023
Gender	—	—	0.059
Male	112 (49.3)	92 (40.5)	—
Female	115 (50.7)	135 (59.5)	—
Stage			
I	95 (41.9)	149 (65.6)	0.000
II	65 (28.6)	40 (17.6)	0.005
III	48 (21.1)	25 (11.0)	0.003
IV	16 (7.0)	8 (3.5)	0.093
T			
T1	57 (25.1)	100 (44.1)	0.000
T2	132 (58.1)	105 (46.3)	0.011
T3	28 (12.3)	11 (4.8)	0.004
T4	9 (4.0)	9 (4.0)	1.000
M			
M0	149 (65.6)	153 (67.4)	0.691
M1	16 (7.0)	7 (3.1)	0.054
N			
N0	129 (56.8)	165 (72.7)	0.000
N1	52 (22.9)	32 (14.1)	0.016
N2	43 (18.9)	20 (8.8)	0.002
N3	1 (0.4)	1 (0.4)	1.000
Fustat	—	—	0.000
Dead	99 (43.6)	57 (25.1)	—
Alive	128 (56.4)	170 (74.9)	—
Futime, years	2 (0.0-18.7)	2.2 (0.0-18.4)	0.006

Data were expressed as mean (min, max) or n (%).

### The Validation in the GEO Cohort

**FIGURE 4:**
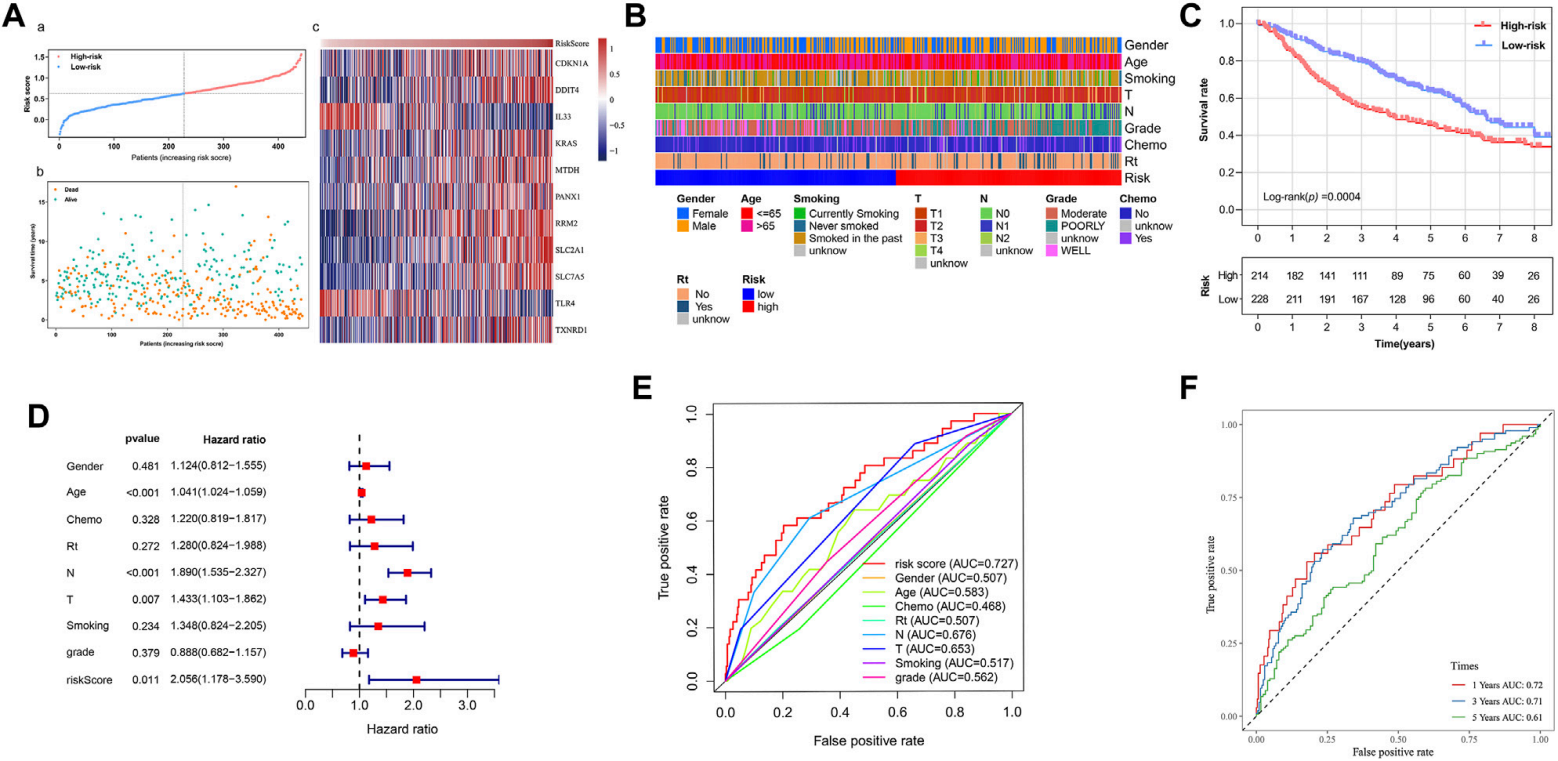
The validation of prognostic signature in the GEO tumor cohort. **(A)** The distribution of survival status, survival time, and risk score and the expression of model genes in two risk groups. **(** **B)** Heatmap of clinicopathological feature distribution in two risk groups. **(** **C)** Kaplan–Meier survival curve of high- and low-risk groups. **(** **D)** Multivariate Cox regression analyses on overall survival. **(** **E)** ROC curves for predicting overall survival of clinical factors and risk score. **(** **F)** ROC curves for predicting the 1-, 3-, and 5-year survival of risk score. ROC: receiver operating characteristic curve. AUC: area under the curve. Chemo: chemotherapy. Rt: radiotherapy.

The data of GEO samples were used to verify the prediction ability of the model. The patients were stratified into a high-risk group (*n* = 214) and a low-risk group (*n* = 228), based on the median value of risk scores of the TCGA tumor cohort. The clinical features of patients in the GSE68465 dataset, such as age, smoking history, chemoradiotherapy, and pathological differentiation, are shown in Table 2. Obviously, risk with survival and gene expression trends were similar to those of the TCGA LUAD cohort (Figure 4A). The heatmap showed the distributions of clinicopathological features in two risk groups (Figure 4B **)**. The significant differences were observed in risk scores within various subgroups classified by clinicopathological features including tumor size stage and lymph node stage (Supplementary Figure S1E–K). Patients with poorer tumor grade or receiving chemotherapy/radiotherapy had significantly higher risk scores. As expected, patients in the high-risk group had a poorer prognosis (Figure 4C, *p* = 0.0004). The results of multivariate Cox regression analysis also verified the independent effective prognostic value of the model (Figure 4D and Supplementary Table S3). Moreover, the AUC value of risk score in the validation cohort was the highest, in line with the result of the training cohort (Figure 4E). In the GEO dataset, the AUC values at 1, 3, and 5 years, respectively, were 0.72, 0.71, and 0.61 (Figure 4F). Taken together, these data indicated that the constructed prognostic tool possessed good performance in predicting survival outcomes.

**TABLE 2:** Characteristics of two risk group patients in GEO LUAD cohort.

Characteristics	GEO-LUAD cohort		
	High-risk	Low-risk	*p* value
	(*n* = 214)	(*n* = 228)	
Age, years	63.7 (33-86)	65.0 (35-87)	0.236
Gender	—	—	0.002
Male	124 (57.9)	99 (43.4)	—
Female	90 (42.1)	129 (56.6)	—
Smoking history			
Yes	152 (71.0)	148 (64.9)	0.169
No	22 (10.3)	35 (15.4)	0.112
Chemotherapy			
Yes	49 (22.9)	40 (17.5)	0.161
No	155 (72.4)	185 (81.1)	0.030
Radiotherapy			
Yes	39 (18.2)	26 (11.4)	0.043
No	165 (77.1)	198 (86.8)	0.008
Differentiation			
Well	11 (5.1)	49 (21.5)	0.000
Moderate	81 (37.9)	128 (56.1)	0.000
Poorly	118 (55.1)	48 (21.1)	0.000
T			
T1	48 (22.4)	102 (44.7)	0.000
T2	140 (65.4)	111 (48.7)	0.000
T3	22 (10.3)	6 (2.6)	0.001
T4	3 (1.4)	8 (3.5)	0.223
N			
N0	135 (63.1)	164 (71.9)	0.047
N1	49 (22.9)	38 (16.7)	0.100
N2	29 (13.6)	24 (10.5)	0.328
Fustat	—	—	0.000
Dead	134 (62.6)	102 (44.7)	—
Alive	80 (37.4)	126 (55.3)	—
Futime, years	4.1 (0.0-17)	4.6 (0.0-13.6)	0.003

Data are expressed as mean (min, max) or n (%).

### GSEA Enrichment Analysis

**FIGURE 5:**
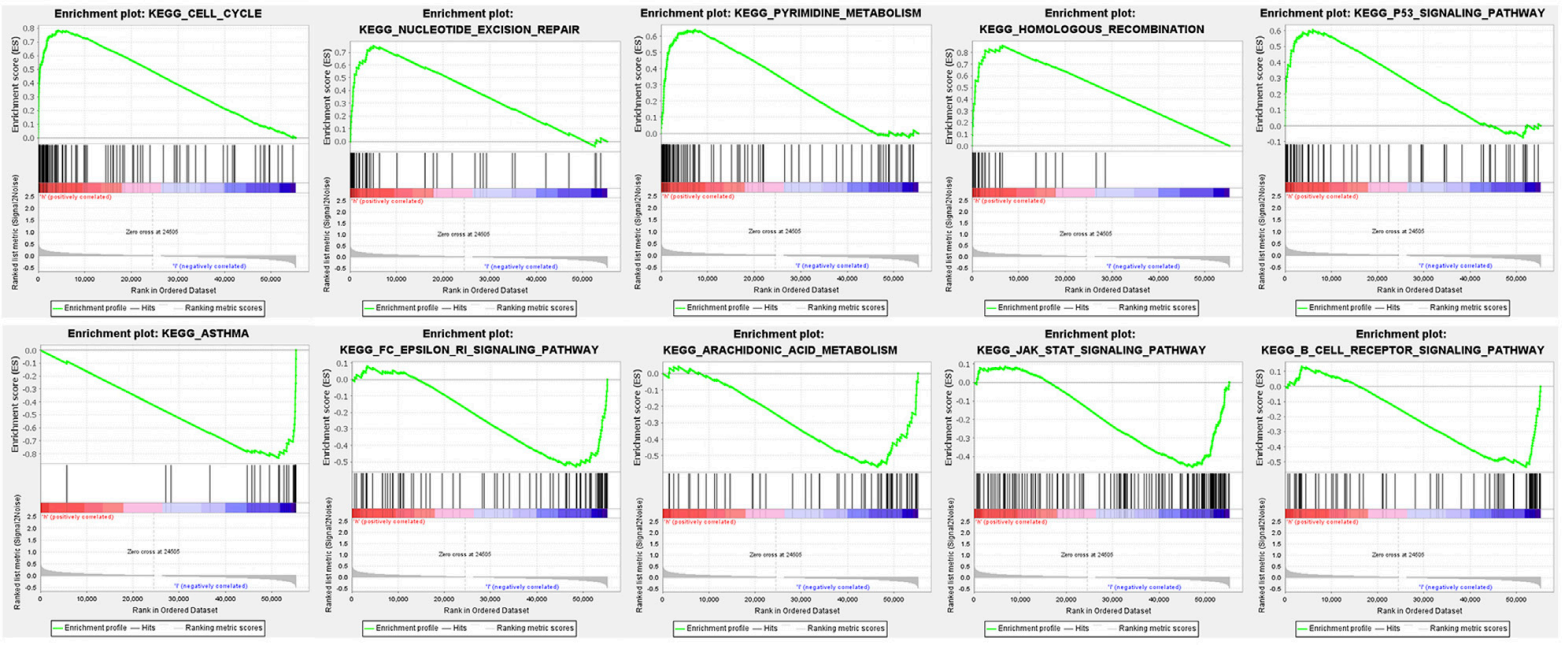
Functional enrichment analysis of genes between high-risk and low-risk groups based on the TCGA tumor dataset.

**FIGURE 6:**
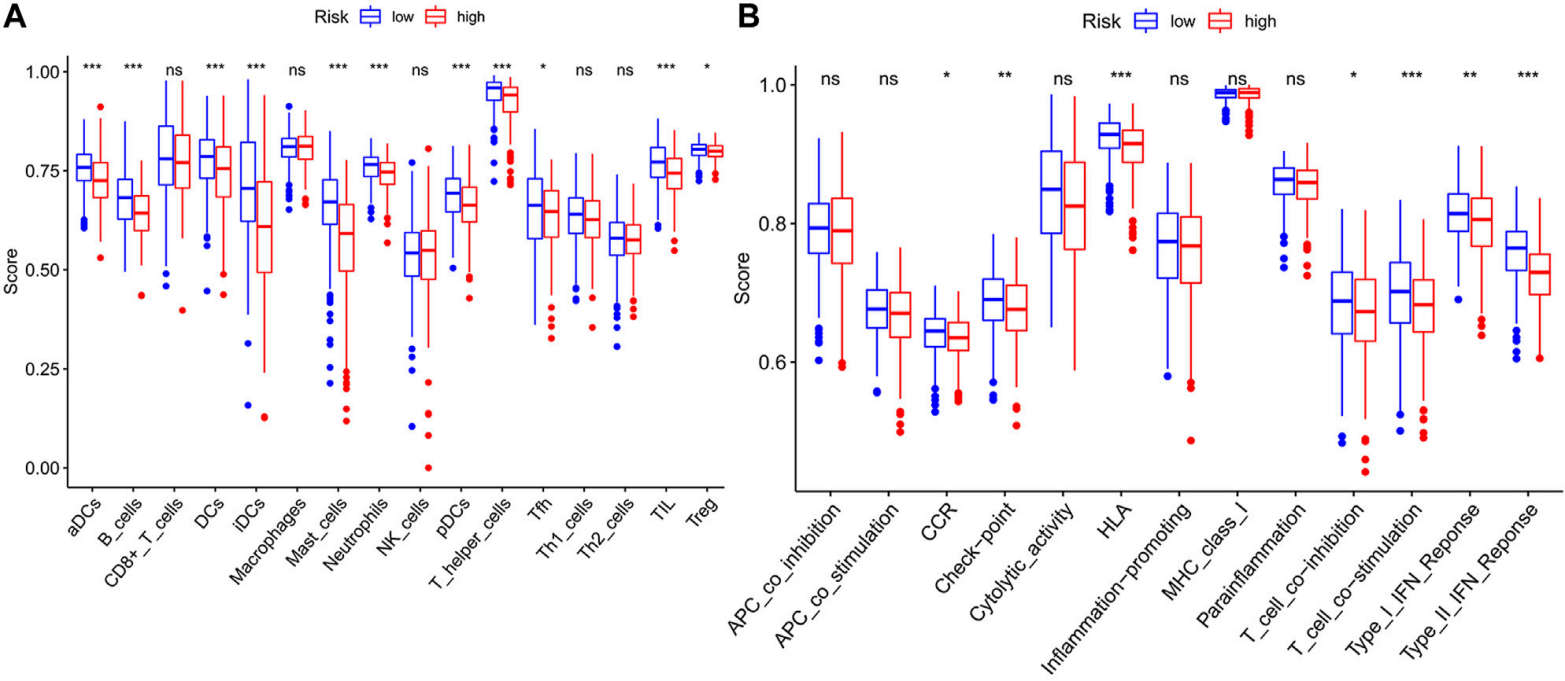
The ssGSEA scores between two risk groups in the TCGA tumor cohort. The scores of 16 immune cells **(A)** and 13 immune-related functions **(B)** are displayed in boxplots. ssGSEA: single-sample gene set enrichment analysis. CCR: cytokine–cytokine receptor. ns, not significant; *p < 0.05; **p < 0.01; ***p < 0.001.

We used GSEA to distinguish the potential functional differences between the two risk groups in the TCGA LUAD cohort. Increased activations of the cell cycle were markedly enriched in the high‐risk group, including pyrimidine metabolism, homologous recombination, and nucleotide excision repair (Figure 5 and Table 3). Inflammation and immune-related pathways such as the Fc epsilon RI-mediated signaling pathway and B cell receptor signaling pathway were enriched in the low‐risk group. The further ssGSEA was performed to score the samples from the high- and low-risk groups in the TCGA tumor cohort, and the differences in immune cells and functions between the groups were detected. The scores of dendritic cells (DCs), B lymphocytes, and T helper cells were significantly higher in the low-risk group, as well as type II IFN response and HLA (Figure 6 **,** *p* < 0.001), which indicated that the low-risk group had greater immune cell infiltration and antitumor immune activities.

**TABLE 3:** Gene functional enrichment in high- and low-risk groups.

Gene set name	Size	NES	NOM *p*-value
KEGG_CELL_CYCLE	125	2.44	0.000
KEGG_PYRIMIDINE_METABOLISM	98	2.27	0.000
KEGG_P53_SIGNALING_PATHWAY	68	2.25	0.000
KEGG_NUCLEOTIDE_EXCISION_REPAIR	44	2.25	0.000
KEGG_HOMOLOGOUS_RECOMBINATION	28	2.18	0.000
KEGG_ASTHMA	28	−2.14	0.002
KEGG_FC_EPSILON_RI_SIGNALING_PATHWAY	79	−2.04	0.002
KEGG_ARACHIDONIC_ACID_METABOLISM	58	−2.01	0.000
KEGG_JAK_STAT_SIGNALING_PATHWAY	155	−1.85	0.010
KEGG_B_CELL_RECEPTOR_SIGNALING_PATHWAY	75	−1.84	0.017

NES, normalized enrichment score; NOM, nominal; Gene sets with NOM p-value <0.05 are considered as significant.

### Online Database Analysis

**FIGURE 7:**
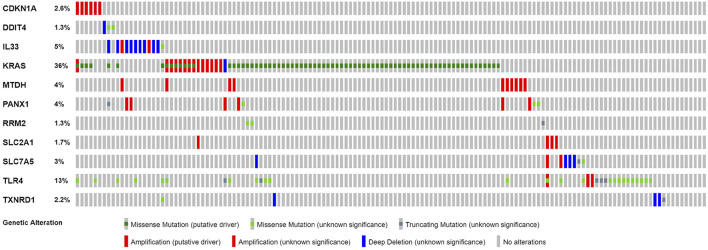
Online database analysis of prognosis ferroptosis-related genes. The total variation frequency of 11 ferroptosis-related genes in LUAD patients.

**FIGURE 8:**
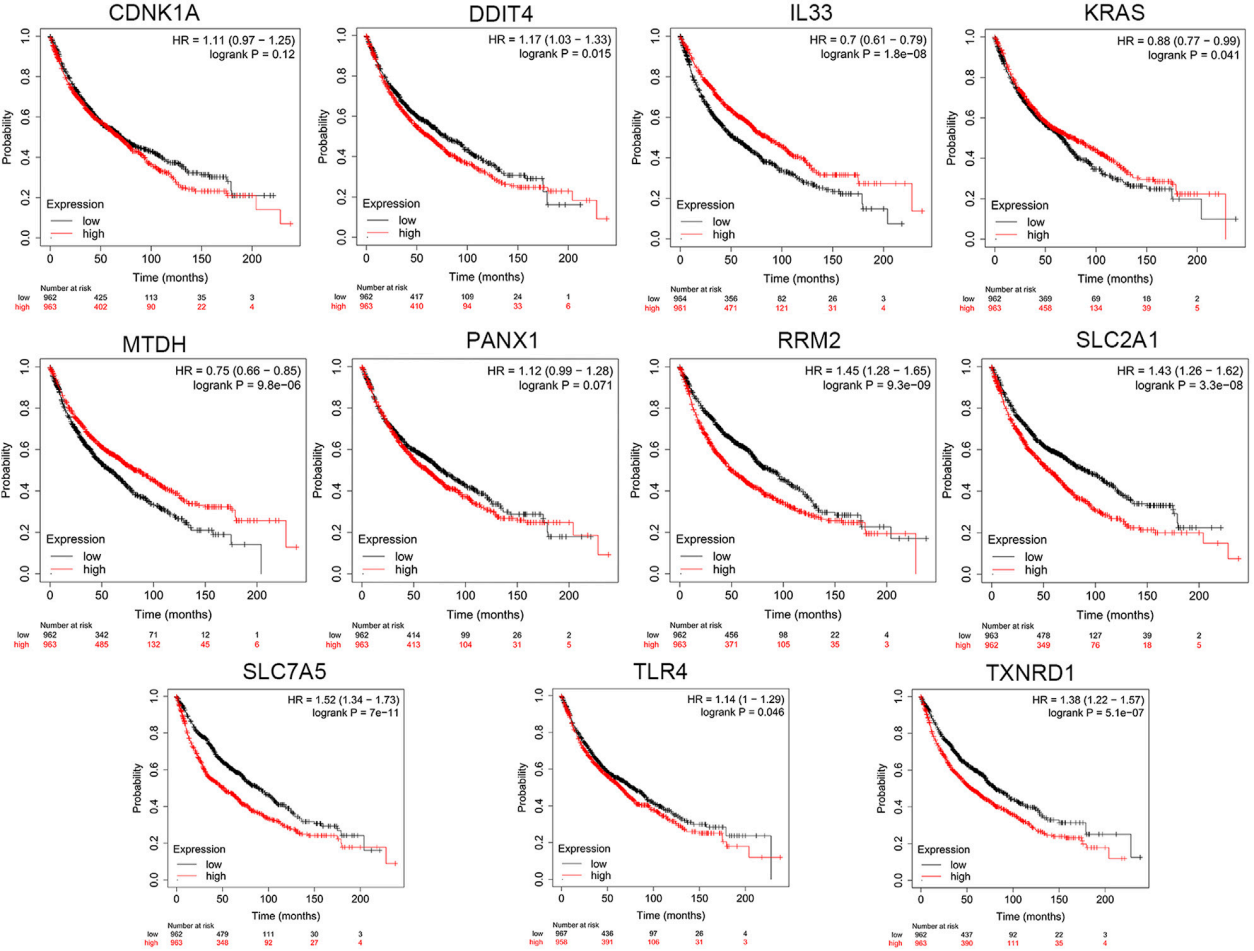
The KM survival analysis between the high and low expressions of 11 ferroptosis-related genes in lung cancer in the online database.

Based on cBioPortal, we explored the frequency and types of mutation in 11 FRGs in LUAD. These genes were altered in 57% of LUAD patients in the online database. KRAS was modified the most, and missense mutations were common (Figure 7). Through the kmplot online tool, KM survival analyses demonstrated that *DDIT4*, *IL33*, *KRAS*, *MTDH*, *RRM2*, *SLC2A1*, *SLC7A5*, *TLR4*, and *TXNRD1* overexpression was markedly related to OS (*p* < 0.05) in lung cancer. The overexpression of *DDIT4*, *RRM2*, *SLC2A1*, *SLC7A5*, *TLR4*, and *TXNRD1* were risk factors for poor prognosis, while *IL33*, *KRAS*, and *MTDH* were the opposite (Figure 8).

## Discussion

LUAD as a highly lethal cancer has a large number of patients worldwide, and only 15% of LUAD patients achieved 5-year survival, despite advances in treatment (Ma et al., 2020). Due to the high heterogeneity and complexity of LUAD, it is still challenging to effectively predict the prognosis of LUAD patients (Song et al., 2019), emphasizing the search for novel biomarkers with good predictive power as well as new treatment targets. Tumor cells can go through several forms of regulated cell death during the cancer development. Ferroptosis has been found participating in the process of cancer cell death, and stimulation of ferroptosis is a hopeful strategy for cancer therapy. Targeted exosome-encapsulated erastin has been demonstrated to efficiently induce ferroptosis in tumor cells (Yu et al., 2019). In our current study, an effective and novel ferroptosis-related prognostic gene signature in LUAD patients was constructed based on the TCGA dataset and was validated in the GEO dataset. Our signature had a good prognostic value and could be used as underlying biomarker and therapeutic target in the ferroptosis regulation pathways.

In this study, we first screened out 86 differentially expressed FRGs and explored their potential functions. The univariate Cox model selected 15 survival‐related DFRGs. Then, we constructed a prognostic 11-FRG signature to predict OS in LUAD patients through LASSO Cox regression analysis. According to our results, *KRAS*, *SL2A1*, *RRM2*, and *TXNRD1* were significantly upregulated genes in LUAD samples. Ribonucleoside-diphosphate reductase subunit M2 (*RRM2*) could promote proliferation and chemotherapy resistance of NSCLC cells *via* upregulating epidermal growth factor receptor expression and AKT phosphorylation (Huang et al., 2019). Recent studies have shown that *RRM2* facilitated tumor immune infiltration through inhibiting ferroptotic death in LUAD patients (Tang et al., 2021). *KRAS* was the most commonly mutated oncogene in lung, pancreatic, and colorectal carcinomas and enabled an improved rate of glutathione regeneration and ferroptosis protection by elevating nicotinamide adenine dinucleotide phosphate hydrogen levels through metabolic reprogramming (Pylayeva-Gupta et al., 2011; Bebber et al., 2020). Solute carrier family two member 1 (*SLC2A1*/*GLUT1*) was an important regulator of the glycolysis pathway and was found to have an increased expression in premalignant lesions and neoplasms of lung cancer patients due to tumors’ high demand of glucose (Ooi and Gomperts, 2015). Jiang et al. indicated that *SLC2A1* inhibited the accumulation of intracellular iron and lipid ROS which were required for ferroptosis (Jiang et al., 2017). Thioredoxin reductase 1 (*TXNRD1*) modulating the cellular redox balance through reducing oxidized thioredoxin (TXN) protected cells against oxidative stress, and direct knockdown of *TNXRD1* increased the basal ROS level and sensitized radiation-resistant lung tumor cells to radiation (Hao et al., 2017). Moreover, the *TXNRD* inhibitor enhanced lysine oxidase (LO)-induced necroptosis and ferroptosis *via* a ROS-dependent mechanism (Chepikova et al., 2020). These aforementioned genes appear to suppress ferroptosis, which may potentially explain the correlation between their high expression and poor prognosis in LUAD patients.

DNA damage-inducible transcript 4 (*DDIT4/REDD1*) was a stress response gene, and its expression increased cellular sensitivity to lethal oxidative stress (Ellisen et al., 2002). The transient elevation of *DDIT4* expression might reduce tumor growth, while high and constitutive expression was linked with poor prognosis in diverse hematologic and solid tumors (Britto et al., 2020). Overexpression of pannexin 1 (*PANX1*) promoted the invasion and migration of hepatocellular carcinoma cells *via* modulation of EMT depending on AKT signaling (Shi et al., 2019). Su et al. indicated that *PANX1* deletion inhibited ferroptinophagy through the MAPK/ERK pathway (Su et al., 2019). Solute carrier family seven member 5 (*SLC7A5*) as an amino acid transporter was overexpressed in multiple cancers including NSCLC, and its expression level was related to cancer progression and aggressiveness (Li et al., 2018). The increased expression of *SLC7A5* facilitated by sublethal concentrations of ferroptosis inducers could facilitate cells better coping with oxidative stress (Alborzinia et al., 2018). Metadherin (*MTDH*) facilitated transcription by regulating transcription factors such as HIF1A and TWIST1 to control cancer cell migration, invasion, and angiogenesis, which was correlated with poor OS in many types of cancers, but it enhanced the vulnerability of tumor cells to ferroptosis through inhibiting *GPX4* and *SLC3A2* (Lu et al., 2018; Bi et al., 2019). It is obvious that these four genes can promote ferroptosis. However, their high expression occurs in many types of tumors and contributes to cancer progression and poor prognosis. Similarly, our study confirmed that these genes were upregulated in LUAD samples and were associated with worse outcomes.

There were also three genes that were significantly downregulated in LUAD tumor tissues. Among them, the expressions of *IL33* and *TLR4* were negatively correlated with poor OS. Interleukin 33 (*IL33*) was an alarmin connected to necroptosis, and its upregulation could be prevented by ferrostatin-1, an inhibitor of ferroptosis (Martin-Sanchez et al., 2017). Kim et al. observed that plasma *IL-33* levels were elevated at the early stage of lung cancer but decreased with advanced stages (Kim et al., 2015). Toll-like receptor 4 (*TLR4*) knockdown could significantly inhibit the ferroptosis through the NADPH oxidase 4 (NOX4) pathway, while *TLR4* signaling activation in LUAD cells activated downstream p65 nucleus translocation and finally promoted proliferation and migration (Zhou et al., 2018; Chen et al., 2019). In addition, *CDKN1A*, cyclin-dependent kinase inhibitor 1A (*CDKN1A*/*p21*), was found to be oncogenic in lung cancer by promoting anti-apoptosis and cell proliferation (Su et al., 2018). Tarangelo et al. reported that *CDKN1A* expression mediated by p53 delayed the onset of ferroptosis induced by cystine deprivation in human cancer cells (Tarangelo et al., 2018). Interestingly, the higher the expression of *CDKN1A*, the higher the risk of poor prognosis, but it was significantly downregulated in tumor tissues. Overall, we think that tumor cells under persistent oxidative stress can make an exquisite balance between the expression of ferroptosis driver genes and suppressor genes, thereby escaping ferroptosis, eventually facilitating proliferation and infiltration. Therefore, understanding the mechanism of these genes acting in ferroptosis may shed new light on treating cancers. Our work explored the effect of these 11 FRGs on tumors, which may provide indispensable sight into future further in-depth research.

In our study, the risk score was calculated based on the eleven meaningful FRGs. We observed a higher risk score related to the clinicopathological characteristics of LUAD patients, such as current smoking history and poor differentiation. Cigarette smoke extract could induce lipid peroxidation and intracellular GSH depletion which are key features of ferroptosis, and ferroptosis played a key role in the toxicity caused by cigarette smoke (Sampilvanjil et al., 2020; Sepand et al., 2021). Tumor progression and resistance to treatment are usually accompanied by the polarization of malignant cells toward a poorly differentiated state, and this transition generates an accumulated vulnerability to the induction of ferroptosis, which may pave the way to novel therapeutic strategies (Chen and Galluzzi, 2018). Here, we also analyzed the potential functional differences after classifying tumor patients into two risk groups according to the risk score. GSEA revealed that tumor-related pathways were most active in the high-risk group, such as the p53 signaling pathway. Tumor suppressor p53 (*TP53*) was frequently mutated in lung cancer, and multiple signaling pathways to induce oncogenicity could be activated by R273H-mutated p53 (Hao et al., 2019). *TP53* was reported to limit ferroptosis by blocking dipeptidyl peptidase-4 activity, although it has been demonstrated to promote ferroptosis, which meant that *TP53* played pleiotropic functions in regulating ferroptosis (Xie et al., 2017). The signal transduction pathway of p53 mediators also had a regulation relationship with our prognostic-related genes *CDKN1A* and *RRM2* (Tarangelo et al., 2018; Jin et al., 2020). In the low-risk group, functional enrichments were mainly linked with immune‐related pathways and the significantly upregulated immune infiltrates including DCs and TILs were also observed. Dendritic cells (DCs) promoting the cross-presentation of tumor-associated antigens were considered paramount in antitumor immunity, and the effector activity of CD8^+^ T cells which were main effectors of anticancer immunity was dependent on DC-derived cytokines (Wculek et al., 2020). Previous studies have suggested that high densities of TILs were correlated with improved OS in multiple tumor types (Liu et al., 2018), and our results reconfirmed this association. Immune-related molecules may play a key role in tumor therapy and may become therapeutic targets. We speculate that the low-risk group patients with a better prognosis benefit from enhanced antitumor immunity. Overall, our prognostic signature may be a reliable tool for risk stratification in LUAD patients.

Ferroptosis as a different form of cell death from autophagy and apoptosis provides tumor treatment with a new therapeutic direction. Nevertheless, cancer cells can exhibit an adaptative response to ferroptosis, and the sensitivity of different tumor cells to ferroptosis may vary greatly. Hence, unveiling the mechanism of ferroptosis resistance versus sensitivity promotion is key to the development of personalized antitumor strategies, and the connection between ferroptosis and host immunogenicity also needs to be explored. In our study, we integrated some ferroptosis biomarkers to predict OS among LUAD patients, which may promote the development of precision medicine in LUAD. Moreover, compared with previous studies and established FRG-related prognostic models (Cai et al., 2021; Wang et al., 2021), our model could provide better predictive performance, and we emphasized the analysis of differentially expressed FRGs and their relationship with OS. However, our study was subject to several limitations. First, all our data were from public databases and our results lacked clinical sample validation. Our signature needs to be proved in independent cohort studies and further experiments of ferroptosis function study in the future. Second, this study failed to explore the underlying link mechanism between ferroptosis and the stages on LUAD development, radiotherapy, and chemotherapy. Third, our research was limited by the comparatively small sample size.

## Conclusion

We defined a novel 11-FRG signature for predicting OS in LUAD patients based on online databases. Our findings may provide useful biomarkers for prognosis prediction and new insights into searching novel molecules or targets for cancer treatment.

## Data Availability

The original contributions presented in the study are included in the article/Supplementary Material; further inquiries can be directed to the corresponding author.
