# Supplementary figures and images for: Establishment and Validation of a Ferroptosis-Related Gene Signature to Predict Overall Survival in Lung Adenocarcinoma
Source: Front Genet. 2022 Jan 14;12:793636. doi: 10.3389/fgene.2021.793636 (PMC8795866; doi:10.3389/fgene.2021.793636)

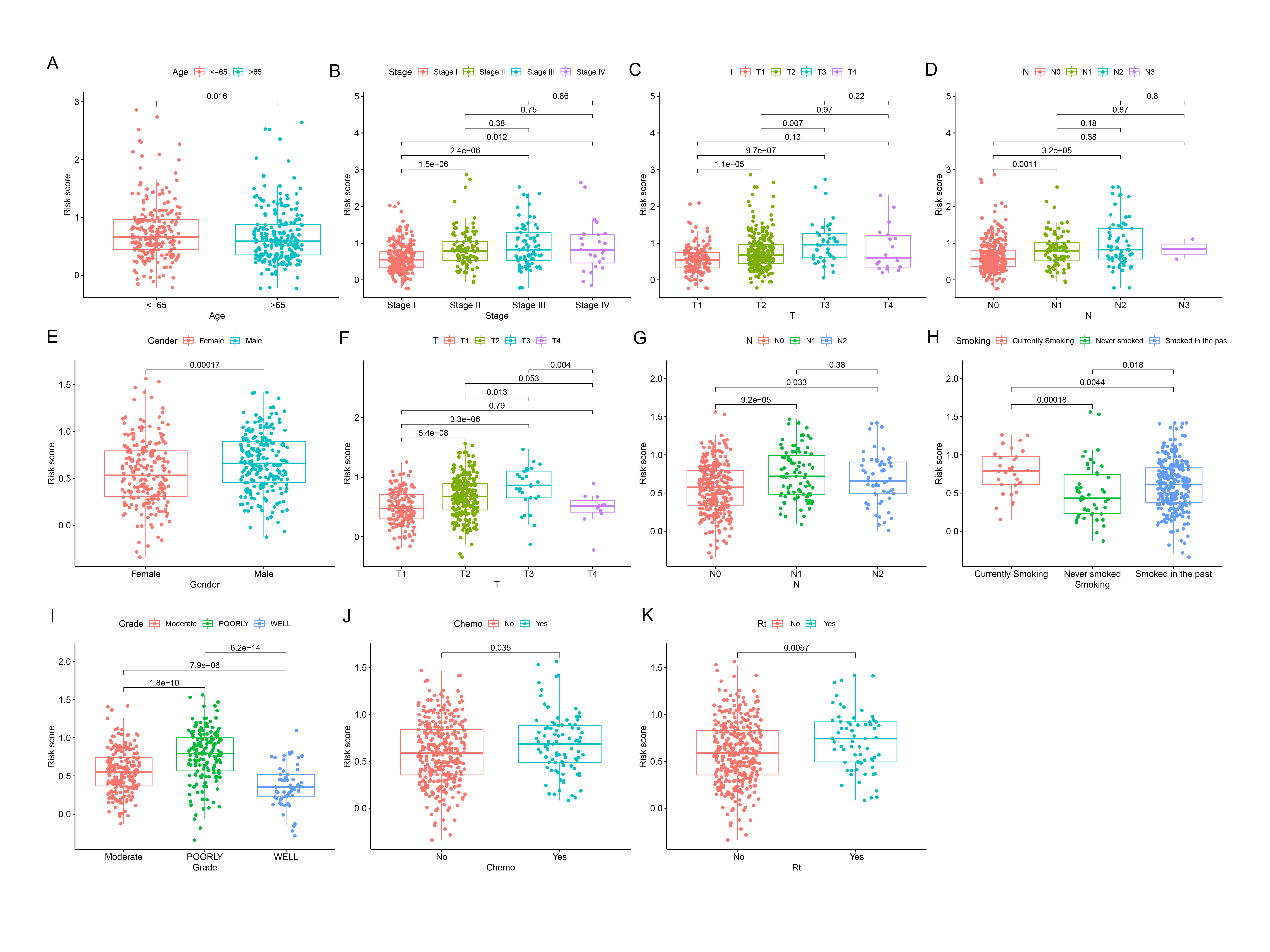

Supplement: Supplementary file 3 [file Image1.JPEG]
