# Supplementary material for: Establishment and Validation of a Ferroptosis-Related Gene Signature to Predict Overall Survival in Lung Adenocarcinoma
Source: Front Genet. 2022 Jan 14;12:793636. doi: 10.3389/fgene.2021.793636 (PMC8795866; doi:10.3389/fgene.2021.793636)
Supplement: Supplementary file 5 [file Table4.DOCX]

**Table 1.** Characteristics of two risk group patients in TCGA LUAD cohort.

| Characteristics | TCGA-LUAD cohort | | |
| --- | --- | --- | --- |
|  | High risk  (n = 227) | Low risk  (n = 227) | P-value |
| Age, years | 63.7 (38-88) | 66.1 (33-87) | 0.023 |
| Gender |  |  | 0.059 |
| Male | 112 (49.3) | 92 (40.5) |  |
| Female | 115 (50.7) | 135 (59.5) |  |
| Stage |  |  |  |
| I | 95 (41.9) | 149 (65.6) | 0.000 |
| II | 65 (28.6) | 40 (17.6) | 0.005 |
| III | 48 (21.1) | 25 (11.0) | 0.003 |
| IV | 16 (7.0) | 8 (3.5) | 0.093 |
| T |  |  |  |
| T1 | 57 (25.1) | 100 (44.1) | 0.000 |
| T2 | 132 (58.1) | 105 (46.3) | 0.011 |
| T3  T4 | 28 (12.3)  9 (4.0) | 11 (4.8)  9 (4.0) | 0.004  1.000 |
| M |  |  |  |
| M0 | 149 (65.6) | 153 (67.4) | 0.691 |
| M1 | 16 (7.0) | 7 (3.1) | 0.054 |
| N |  |  |  |
| N0 | 129 (56.8) | 165 (72.7) | 0.000 |
| N1 | 52 (22.9) | 32 (14.1) | 0.016 |
| N2 | 43 (18.9) | 20 (8.8) | 0.002 |
| N3 | 1 (0.4) | 1 (0.4) | 1.000 |
| Fustat |  |  | 0.000 |
| Dead | 99 (43.6) | 57 (25.1) |  |
| Alive | 128 (56.4) | 170 (74.9) |  |
| Futime, years | 2 (0.0-18.7) | 2.2 (0.0-18.4) | 0.006 |

Data were expressed as mean (min, max) or n (%).

**Table 2.** Characteristics of two risk group patients in GEO LUAD cohort.

| Characteristics | GEO-LUAD cohort | | |
| --- | --- | --- | --- |
|  | High-risk  (n = 214) | Low-risk  (n = 228) | P value |
| Age, years | 63.7 (33-86) | 65.0 (35-87) | 0.236 |
| Gender |  |  | 0.002 |
| Male | 124 (57.9) | 99 (43.4) |  |
| Female | 90 (42.1) | 129 (56.6) |  |
| Smoking history |  |  |  |
| Yes | 152 (71.0) | 148 (64.9) | 0.169 |
| No | 22 (10.3) | 35 (15.4) | 0.112 |
| Chemotherapy |  |  |  |
| Yes | 49 (22.9) | 40 (17.5) | 0.161 |
| No | 155 (72.4) | 185 (81.1) | 0.030 |
| Radiotherapy |  |  |  |
| Yes | 39 (18.2) | 26 (11.4) | 0.043 |
| No | 165 (77.1) | 198 (86.8) | 0.008 |
| Differentiation |  |  |  |
| Well | 11 (5.1) | 49 (21.5) | 0.000 |
| Moderate | 81 (37.9) | 128 (56.1) | 0.000 |
| Poorly | 118 (55.1) | 48 (21.1) | 0.000 |
| T |  |  |  |
| T1 | 48 (22.4) | 102 (44.7) | 0.000 |
| T2 | 140 (65.4) | 111 (48.7) | 0.000 |
| T3 | 22 (10.3) | 6 (2.6) | 0.001 |
| T4 | 3 (1.4) | 8 (3.5) | 0.223 |
| N |  |  |  |
| N0 | 135 (63.1) | 164 (71.9) | 0.047 |
| N1 | 49 (22.9) | 38 (16.7) | 0.100 |
| N2 | 29 (13.6) | 24 (10.5) | 0.328 |
| Fustat |  |  | 0.000 |
| Dead | 134 (62.6) | 102 (44.7) |  |
| Alive | 80 (37.4) | 126 (55.3) |  |
| Futime , years | 4.1 (0.0-17) | 4.6 (0.0 -13.6) | 0.003 |

Data are expressed as mean (min, max) or n (%).

| Gene set name | SIZE | NES | NOM P-value |
| --- | --- | --- | --- |
| KEGG_CELL_CYCLE | 125 | 2.44 | 0.000 |
| KEGG_PYRIMIDINE_METABOLISM | 98 | 2.27 | 0.000 |
| KEGG_P53_SIGNALING_PATHWAY | 68 | 2.25 | 0.000 |
| KEGG_NUCLEOTIDE_EXCISION_REPAIR | 44 | 2.25 | 0.000 |
| KEGG_HOMOLOGOUS_RECOMBINATION | 28 | 2.18 | 0.000 |
| KEGG_ASTHMA | 28 | -2.14 | 0.002 |
| KEGG_FC_EPSILON_RI_SIGNALING_PATHWAY | 79 | -2.04 | 0.002 |
| KEGG_ARACHIDONIC_ACID_METABOLISM | 58 | -2.01 | 0.000 |
| KEGG_JAK_STAT_SIGNALING_PATHWAY | 155 | -1.85 | 0.010 |
| KEGG_B_CELL_RECEPTOR_SIGNALING_PATHWAY | 75 | -1.84 | 0.017 |

**Table 3**. Gene functional enrichment in high- and low-risk groups.

NES: normalized enrichment score; NOM: nominal; Gene sets with NOM P-value <0.05 are considered as significant.
